# Comprehensive Analysis of Mitochondrial Genomic Characteristics and Phylogenetic Analysis of the Plant Genus *Ipomoea* (Convolvulaceae) Species

**DOI:** 10.3390/biology14121696

**Published:** 2025-11-28

**Authors:** Mengya Xiao, Cheng Zhang, Wenbang Hou, Youjun Li

**Affiliations:** 1 College of Agriculture, Henan University of Science and Technology, Luoyang 471023, China; 2 College of Animal Science and Technology, Henan University of Science and Technology, Luoyang 471000, China; 3 Sweet Potato Industry Institute, Henan University of Science and Technology, Luoyang 471023, China

**Keywords:** *Ipomoea*, mitogenomes, phylogenetics, genomic evolution

## Abstract

The genus *Ipomoea* holds significant economic and ecological value, yet comparative genomic studies on its mitochondrial genomes remain limited. To clarify the genetic evolutionary characteristics of *Ipomoea* at the mitogenome level, this study conducted a systematic comparative analysis of eight *Ipomoea* mitogenomes, focusing on sequence structure, codon usage bias, repetitive sequences, genetic variation, and phylogenetic relationships. The results showed that the mitogenome sizes of the eight species ranged from 106.281 to 296.652 kb, with GC content of 43.89–44.82%, ORF numbers of 298–326, and RNA editing sites of 29–223. Repetitive sequence analysis revealed universal mononucleotide SSRs (P1), lineage-specific dispersed repeats (DNA/MIR in four species), and abundant short tandem repeats (0–50 bp). Ka/Ks ratio analysis showed 17 protein-coding genes (e.g., *atp4*, *nad6*) under positive selection (linked to adaptive evolution) and 23 genes (e.g., *nad3*) under purifying selection, ensuring functional conservation. Maximum likelihood phylogeny (with *Cuscuta australis* as outgroup) identified three primary clades: *I. quamoclit*–*I. pes-caprae*, *I. triloba*–*I. batatas*, and *I. biflora*–*I. aquatica*, with *I. trifida* and *I. nil* as basal lineages. This study fills gaps in *Ipomoea* mitogenome research and provides a theoretical foundation for understanding its genomic evolution and guiding germplasm improvement.

## Simple Summary

This study explored the genetic material inside the energy-producing parts of cells, known as mitochondrial genomes, in eight different species of the *Ipomoea* plant. Although these plants are valuable for food and the environment, little was known about their mitochondrial genomes. Our goal was to understand how these genomes are structured and how they have evolved. We found that the size and gene content of these genomes vary widely among species. Some genes are changing quickly, which may help the plants adapt to their environments, while others remain very stable. By comparing all the genomes, we were able to map out the family relationships between the species. This research provides a foundation for better understanding how these plants evolve and can help guide future efforts to improve crop varieties, which is important for ensuring food security and sustainable agriculture.

## 1. Introduction

The genus *Ipomoea*, belonging to the order Solanales and the family Convolvulaceae, is the largest genus within its family, comprising approximately 500 species. Most of these species are distributed across subtropical and tropical regions and are predominantly annual or perennial herbaceous vines. Commonly known examples include morning glory (*I. nil*), sweet potato (*I. batatas*), water spinach (*I. aquatica*), and cypress vine (*I. quamoclit*), while a small number are shrubs or small trees [1,2]. In China, about 20 species of *Ipomoea* are found throughout the country, with the majority occurring in southern and southwestern regions [3]. Among these, *I. batatas* and *I. aquatica*. serve as important food and vegetable crops, while some species also possess ornamental value [4]. Additionally, *Ipomoea* species constitute major components of vegetation across various habitats, underscoring their significant economic and ecological importance.

Within plant cells, mitochondria serve as the primary energy-producing organelles, supplying the necessary energy for diverse cellular activities. Since the completion of the *Arabidopsis thaliana* mitochondrial genome sequencing in 1997, the NCBI-Genome database has included over 560 plant mitochondrial genomes to date [5,6]. Unlike animal mitochondrial genomes, plant mitochondrial genomes exhibit considerable variation in size, sequence composition, and the arrangement of functional genes [7,8]. The smallest plant mitochondrial genome known is that of *Brassica napus*, measuring only 221 kb, while the largest, reported to date, is that of *Silene conoidea*, with a genome size of 11.3 Mb [9]. Despite the substantial divergence in genome size among plant mitochondria, most protein-coding genes are highly conserved. These genes primarily consist of 24 core conserved genes and 17 variable genes, which can be categorized based on the types of proteins they encode into Complex I (*nad*), Complex II (*sdh*), Complex III (*cob*), Complex IV (*cox*), Complex V (*atp*), Cytochrome c biosynthesis genes (*ccm*), and transfer RNA (tRNA) genes, among others [10,11]. In higher plant mitochondrial genomes, genes other than those encoding Complex II, ribosomal proteins, and tRNAs are relatively conserved [12]. Unlike chloroplast genomes, plant mitochondrial genomes do not utilize unique genetic codes, plant mitochondrial genomes share a universal genetic code across species. In addition to being directly inherited from ancestral mitochondria, plant mitochondrial tRNAs also originate from the transfer of sequences from their own chloroplast genomes [13,14]. Due to the lack of a complete set of tRNA genes sufficient to recognize all codons, nearly all higher plants lack specific tRNA genes capable of recognizing Gly (glycine), Val (valine), Leu (leucine), Ala (alanine), Thr (threonine), and Arg (arginine) [15,16]. The coding genes in higher plant mitochondrial genomes also harbor abundant genetic variation information, making them ideal molecular markers for studying species origin, evolution, and population genetic diversity [17]. Mitochondrial genomes not only exhibit rapid evolutionary rates and high recombination rates but also possess advantages such as relatively small genome sizes and ease of sequencing, making them valuable tools for comparative genetics and phylogenetic studies across different plant species [18,19,20].

To date, studies employing comparative genomic approaches to analyze the genomic characteristics and phylogenetic relationships of mitochondrial genomes within this genus remain scarce. To gain deeper insights into the genetic evolutionary characteristics among *Ipomoea* species at the mitochondrial genome level, this study conducts a comparative analysis based on the nine available mitochondrial genomes of *Ipomoea* species in the NCBI-Genome database. The analysis focuses on sequence structure characteristics, codon usage bias, and phylogenetic relationships within the genus. The findings aim to provide a theoretical foundation for a comprehensive understanding of the genomic structural features and phylogenetic diversity among *Ipomoea* species while also laying the groundwork for future omics studies, biological functional analyses, and genomic evolutionary research in this genus.

## 2. Materials and Methods

### 2.1. Sample Source

Experimental samples were collected from the *I. batatas* trial field in Fandian Town, Luoyang City, Henan Province. Fresh and tender leaves in the vigorous growth stage were harvested, rinsed to remove soil and sand, treated with sterile water, and subsequently stored at -80 °C in an ultra-low temperature freezer for future use.

### 2.2. Sequencing

Total genomic DNA was extracted from the tissue using the modified CTAB method [21]. Second-generation sequencing (SGS) and third-generation sequencing (TGS) were performed utilizing the BGISEQ-500 platform and the BGI PromethION nanopore sequencer, respectively. The SGS workflow encompassed sample quality control, library construction, library quality assessment, and sequencing. The TGS procedure involved the following steps: large-fragment DNA was size-selected using the Blue Pippin automated nucleic acid recovery system; libraries were constructed; approximately 1 μg of DNA from each Blue Pippin-selected library was subjected to damage repair; the repaired DNA was purified and recovered using magnetic beads; its concentration was quantified and the recovery rate calculated using a Qubit fluorometer (ThermoFisher Scientific, Waltham, MA, USA); DNA was ligated to sequencing adapters provided in the kit, ensuring each DNA fragment contained identifiable unique adapter sequences; the ligated sample DNA was purified again using magnetic beads; its concentration was measured and the recovery rate calculated; finally, the libraries were loaded onto the sequencer for sequencing.

### 2.3. Mitochondrial Genome Assembly and Annotation

Initially, a 20 G dataset was extracted from the raw PacBio sequencing data using the ‘-s’ and ‘-p’ parameters of the seqkit software (https://github.com/shenwei356/seqkit accessed on 15 May 2025), followed by de novo assembly using Flye (v2.9.3-b1797) [22]. Subsequently, the assembled scaffold sequences were aligned using BLAST (https://blast.ncbi.nlm.nih.gov/Blast.cgi, accessed on 12 June 2025) to identify the draft mitochondrial genome of *I. batatas*. To enhance assembly quality, Samtools (v1.13) was employed to calculate coverage depth, and scaffolds with coverage greater than 20× were selected. Finally, a local database of the assembled sequences was constructed using the ‘makeblastdb’ command in BLAST, and conserved contig sequences were identified via BLAST to finalize the assembly. The mitochondrial genome of *I. batatas*. was annotated using the GeSeq website (https://chlorobox.mpimp-golm.mpg.de/geseq.html, accessed on 12 June 2025), with both the “Protein search identity” and “rRNA, tRNA, DNA search identity” parameters set to 85. Genes with suboptimal annotation results were manually corrected to ensure accuracy and completeness.

### 2.4. Analysis of Mitochondrial Genome Structural Features

The sequenced mitochondrial genome of *I. batatas*. along with mitochondrial genome data from seven other known *Ipomoea* species (that is, *I. batatas* (OM808941.1), *I. biflora* (MZ240723.1), *I. nil* (AP017303.1), *I. pes-caprae* (MZ240736.1), *I. quamoclit* (MZ240732.1), *I. trifida* (LC773296.1), *I. triloba* (BK059242.1)) were imported sequentially into Vector NTI 8.0 software. The imported sequence files were manipulated in different windows to statistically analyze structural characteristics, including mitochondrial genome size, (G + C) content, and the number of ORFs for each species. Editing sites in protein-coding genes were predicted using the online tool PREPACT (http://www.prepact.de/prepact-main.php, accessed on 18 June 2025), with the parameter threshold set to 0.8. Simple sequence repeats (SSRs), dispersed repeats, and tandem repeats were identified and analyzed using the tools MISA-web (https://webblast.ipk-gatersleben.de/misa/, accessed on 18 June 2025; parameters: 1-10, 2-6, 3-5, 4-5, 5-5, 6-5, and C-100), RepeatMasker (https://www.repeatmasker.org/; accessed on 18 June 2025, parameters: default), and Tandem Repeats Finder (https://tandem.bu.edu/trf/home; accessed on 18 June 2025; parameters: 2-7-7, 50, 500, 2-f-d-m), respectively.

### 2.5. Codon Usage Bias Analysis of Mitochondrial Protein-Coding Genes

The RSCU (Relative Synonymous Codon Usage) values were statistically estimated using CodonW software to evaluate the usage bias of each synonymous codon. The RSCU value is defined as the ratio of the observed frequency of a codon to its expected frequency within its synonymous codon family [23]. An RSCU value equal to 1 indicates no usage bias for that synonymous codon, meaning its usage frequency is close to the expected frequency. A codon with usage bias exhibits a higher (or lower) observed frequency than expected, resulting in an RSCU value greater than (or less than) 1.

### 2.6. Analysis of Genetic Variation in the Mitochondrial Genome

Multiple sequence alignment of conserved protein-coding gene sequences from the mitochondrial genomes of eight *Ipomoea* species was performed using Clustal X software. Genetic variation information, including the non-synonymous substitution rate (Ka) and synonymous substitution rate (Ks) for these conserved protein-coding genes across different species’ mitochondrial genomes, was calculated using KaKs_Calculator 3.0 software. Finally, the Ka/Ks ratio for each conserved protein-coding gene was summarized.

### 2.7. Phylogenetic Analysis

The multiple sequence alignment results of the conserved protein-coding gene sequences from the mitochondrial genomes of the eight *Ipomoea* species were imported into MEGA11 software to construct a phylogenetic tree.

## 3. Results

### 3.1. Characteristics of Mitochondrial Genomes in Ipomoea Species

The mitochondrial genome sizes of the eight *Ipomoea* species were observed to range from 106.281 to 296.652 kb, with the largest mitochondrial genome being identified in *I. aquatica* and the smallest in *I. biflora* (Table 1). The guanine-plus-cytosine (G + C) content of the mitochondrial genomes across these eight *Ipomoea* species was found to vary between 43.89% and 44.82%. The highest G + C content was exhibited by *I. pes-caprae*, while the lowest was recorded in *I. triloba*. A shared G + C content of 44.25% was detected in both *I. quamoclit* and *I. batatas*. An open reading frame (ORF) is defined as a DNA sequence that begins with a start codon and ends with a stop codon (excluding the stop codon itself), possessing the potential to encode a protein. The number of ORFs identified in the mitochondrial genomes of the eight *Ipomoea* species varied from 298 to 326. The highest number of ORFs was contained in *I. quamoclit*, whereas *I. triloba* was found to contain 298 ORFs. A total of 324 ORFs were identified in each of *I. biflora* and *I. nil*. Analysis of RNA editing sites, based on the protein-coding gene (PCG) sequences from these eight mitochondrial genomes, revealed that more than 200 RNA editing sites were present in *I. quamoclit*, *I. biflora*, *I. batatas*, and *I. trifida*. Among these, the highest number of RNA editing sites (223) was possessed by *I. trifida*. Between 150 and 200 RNA editing sites were identified in *I. aquatica*, *I. nil*, and *I. triloba*. In contrast, the number of RNA editing sites in *I. pes-caprae* is relatively small, with only 29.

### 3.2. Characteristics of Protein-Coding Genes in the Mitochondrial Genomes of the Genus Ipomoea

**Figure 1:**
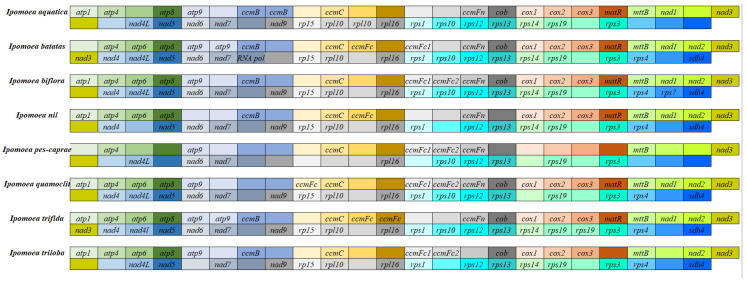
The composition of mitochondrial protein-coding genes of the eight *Ipomoea* mitochondrion.

Variations in size and (G + C) content were observed among the mitochondrial genomes of plants within the genus *Ipomoea*. Differences in the products of protein-coding genes were also identified across these mitochondrial genomes, which were found to encode a total of 17 to 40 proteins (Figure 1). The protein-coding genes identified in the eight *Ipomoea* mitochondrial genomes were categorized as follows: Complex I genes, specifically NADH dehydrogenase genes (*nad1*–*nad7*, *nad4L*, and *nad9*); a Complex II gene, the membrane anchor subunit 4 (*sdh4*); a Complex III gene, the cytochrome reductase gene (*cob*); Complex IV genes, namely cytochrome oxidase genes (*cox1*–*cox3*); Complex V genes, the ATP synthase genes (*atp1*, *atp4*, *atp6*, *atp8*, and *atp9*); Cytochrome C biogenesis genes (*ccmB*, *ccmC*, *ccmFC1*, *ccmFC2*, and *ccmFN*); ribosomal protein genes (e.g., *rpl*, *rps*); the SecY-dependent transporter gene (*mttB*); and the intron maturase gene (*matR*). Among these, the mitochondrial genome of *I. trifida* was found to contain the highest diversity of protein-coding genes, with 40 types identified. Conversely, the mitochondrial genome of *I. pes-caprae* contained the fewest, with only 17 types. Variability in protein-coding genes was also noted among the different mitochondrial genomes. Twelve genes, including *rpl5*, *rpl10*, *rpl16*, and *rps14*, exhibited relatively high variability, with the *rps12-14* region being particularly variable. The genes *rps12* and *rps13* were present in the mitochondrial genomes of all eight *Ipomoea* species, whereas *rps14* was absent in *I. pes-caprae* and *I. quamoclit* but present in the other six species. Furthermore, multiple-copy phenomena for specific protein-coding genes were observed within the mitochondrial genomes of different *Ipomoea* species. For instance, single copies of *atp9* and *nad3* were identified in the mitochondrial genomes of *I. batatas* and *I. trifida*. In contrast, no copies of these genes were detected in the mitochondrial genomes of *I. biflora*, *I. nil*, *I. pes-caprae*, *I. quamoclit*, and *I. triloba*.

The amino acid preferences of the protein-coding genes were found to be similar across the mitochondrial genomes of the different *Ipomoea* species. All protein-coding genes in the mitochondria of every species were observed to encode 20 amino acids, including isoleucine (Ile), methionine (Met), and proline (Pro). However, the Relative Synonymous Codon Usage (RSCU) for the same protein-coding gene differed among the eight *Ipomoea* mitochondrial genomes. Among the various codons used for the 20 amino acids, GUU (Valine) and GCU (Alanine) were identified as the codons with relatively high usage frequencies. With the exception of *I. nil*, for which GUU was found to have the highest RSCU value, GCU was identified as the codon with the highest RSCU in each of the remaining *Ipomoea* species. In contrast, codons with relatively low RSCU values included UAG (Stop), UAC (Tyrosine), CAC (Histidine), CAG (Glutamine), GGC (Glycine), GAC (Aspartic acid), and CGC (Arginine). The RSCU values for these codons ranged from 0.36 to 0.63.

### 3.3. Analysis of Mitochondrial Genome tRNAs in Ipomoea Species

**Figure 2:**
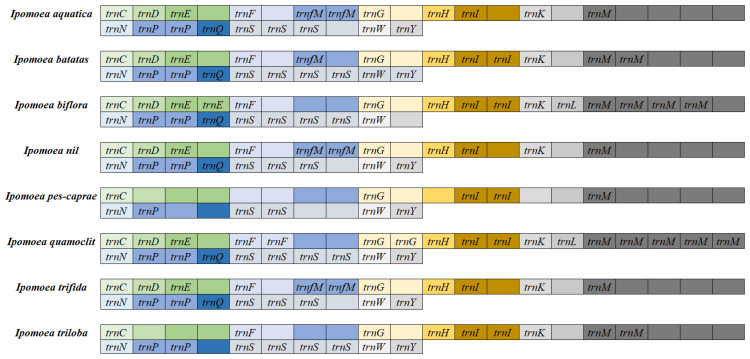
The information of tRNAs in the eight *Ipomoea* mitochondrion.

Among the mitochondrial genomes of the eight *Ipomoea* species, *I. quamoclit* was identified as containing the highest diversity of tRNAs, with 27 distinct types; conversely, *I. pes-caprae* possessed the fewest, with only 11 types. The mitochondrial genomes of *I. nil* and *I. trifida* were found to contain an identical number of tRNA types, each comprising 20. With the exception of *I. pes-caprae*, the mitochondrial genomes of the other seven *Ipomoea* species were observed to contain *trnC*, *trnF*, *trnG*, *trnH*, *trnK*, *trnM*, *trnN*, *trnS*, *trnW*, and *trnY*, which were considered relatively conserved tRNA types (Figure 2). In contrast, *trnI* and *trnP* exhibited considerable variability among the Ipomoea mitochondrial genomes, with *trnP* demonstrating the highest degree of variability. Multiple copies of the same tRNA type were also detected across different *Ipomoea* mitochondrial genomes. Except in *I. nil* and *I. trifida*, *trnI* was present as a single copy in the mitochondrial genomes of the other six *Ipomoea* species. The *trnS* gene was identified with three copies in the mitochondrial genomes of *I. aquatica*, *I. biflora*, *I. quamoclit*, and *I. triloba*, while two copies were observed in *I. nil* and *I. trifida*, and only one copy was found in *I. pes-caprae*. Regarding *trnM*, the highest copy number was recorded in the mitochondrial genome of *I. quamoclit*, with four copies. Two copies were detected in *I. biflora*, and one copy each was identified in *I. aquatica* and *I. triloba*. No copies were observed in the remaining species (Figure 2).

### 3.4. Analysis of Repetitive Sequences in the Mitochondrial Genomes of the Ipomoea Species

**Figure 3:**
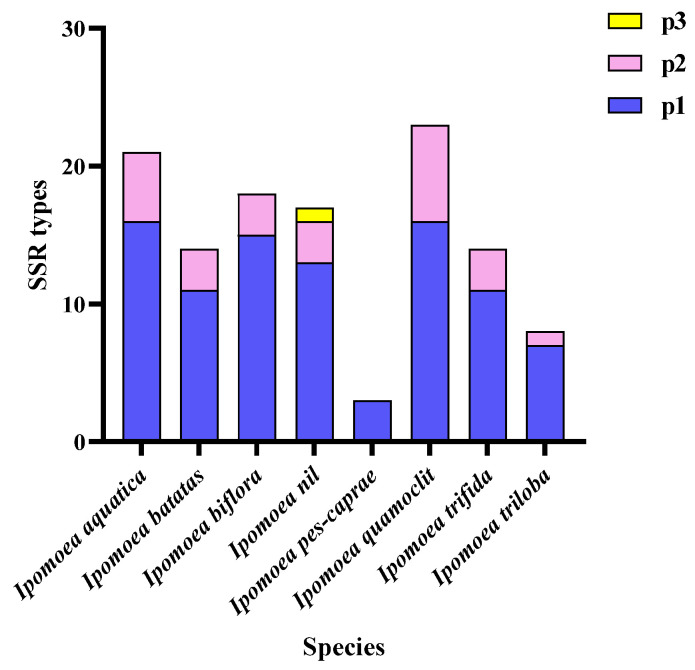
Composition of SSR types in the eight *Ipomoea* mitochondrion.

**Figure 4:**
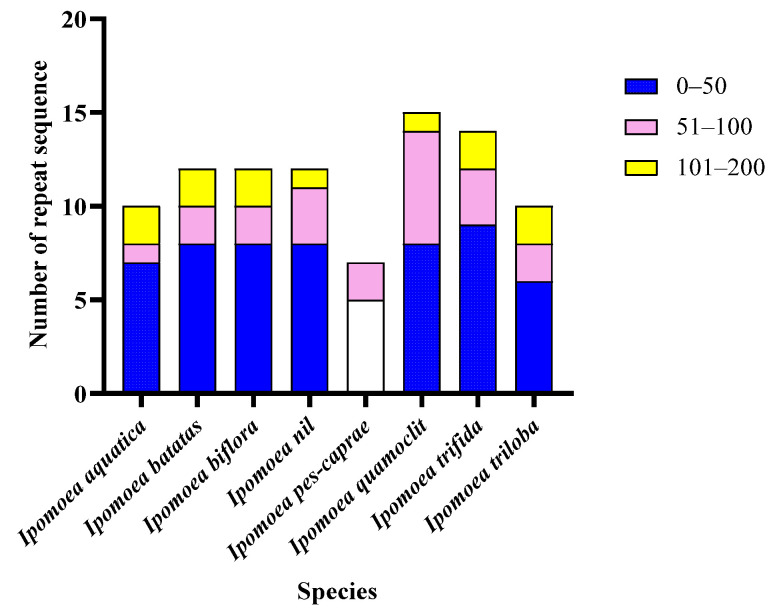
Composition of the number of repeat sequence in the eight *Ipomoea* mitochondrion.

The types and quantities of simple sequence repeats (SSRs), interspersed repeats, and tandem repeats present in the mitochondrial genomes of the eight *Ipomoea* species are illustrated in Figure 3. Three types of SSRs—P1 (mononucleotide), P2 (dinucleotide), and P3 (trinucleotide)—were identified. Among these, P1 was detected in all eight mitochondrial genomes, whereas P3 was exclusively found in the mitochondrial genome of *I. nil*. Variations in the number of SSRs were observed among the mitochondrial genomes of different species. Although P1 was the most abundant type of SSR in all eight species, the maximum number of P2 was recorded in *I. quamoclit* (7), followed by *I. aquatica* (5). In contrast, only one P2 was identified in *I. triloba*, and no P2 were detected in *I. pes-caprae*. The remaining four *Ipomoea* species each contained three P2. The total number of interspersed repeats ranged from 41 in *I. triloba* to 353 in *I. trifida*. DNA/MIR elements were exclusively identified in the mitochondrial genomes of *I. biflora*, *I. nil*, *I. quamoclit*, and *I. trifida*. Similarly, L3/CR1 elements were only detected in *I. trifida*, *I. pes-caprae*, and *I. biflora*. Tandem repeat analysis revealed three length categories: 0–50 bp, 51–100 bp, and 101–200 bp. Tandem repeats within the 0–50 bp were present in all eight species and exhibited the highest relative abundance. The 51–100 bp category was most frequent in *I. quamoclit* (6), followed by *I. trifida* (3), *I. aquatica* (1), and two repeats in each of the remaining five species. In the 101–200 bp category, one repeat was found in *I. quamoclit* and *I. nil*, whereas none were detected in *I. pes-caprae*. The other five species each contained two repeats in this size range (Figure 4).

### 3.5. Genetic Variation Information in Mitochondrial Genomes of Ipomoea Species

To determine the conservation of protein-coding genes across different *Ipomoea* species, the ratio of nonsynonymous substitution rate (Ka) to synonymous substitution rate (Ks) was used to analyze the evolutionary rates of protein-coding genes in the mitochondrial genomes of *Ipomoea* species (Table 2). It was found that *atp4*, *atp8*, *atp9*, *ccmFc*, *ccmFc1*, *ccmFc2*, *ccmFn*, *mttB*, *nad1*, *nad2*, *nad6*, *NADH*, rpl5, *rpl10*, *rps4*, *rps14*, and *sdh4* were genes with relatively rapid evolutionary rates, as their Ka/Ks ratios all exceeded 1. This indicated that these genes were subjected to varying degrees of positive selection. In contrast, the remaining 23 protein-coding genes exhibited Ka/Ks ratios below 1 (ranging from 0.7135 to 0.9878), with *nad3* identified as the most slowly evolving gene. These findings suggested that these genes were influenced by varying degrees of negative selection (purifying selection). Harmful mutations were eliminated through selection, and protein sequences were conserved, thereby reflecting the evolutionary conservatism of *Ipomoea* species.

### 3.6. Phylogenetic Analysis

**Figure 5:**
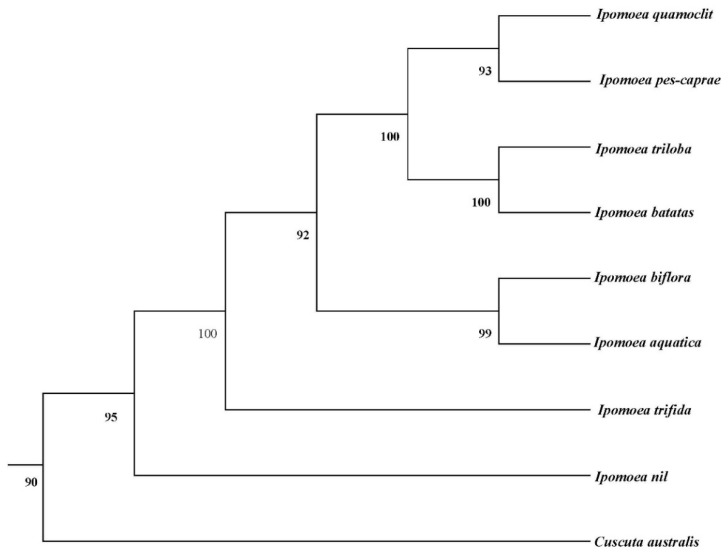
The phylogenetic tree of *eight Ipomoea* mitochondrion based on the protein-coding genes.

A maximum likelihood (ML) tree was constructed using the protein-coding gene (PCG) sequences from eight mitochondrial genomes of *Ipomoea* species, with *Cuscuta australis* (MN400576.1) of the same family but a different genus designated as the outgroup. The optimal substitution model was determined by ModelFinder to be GY-HKY. Based on 1000 bootstrap replicates, the ML phylogenetic tree revealed that *I. quamoclit* and *I. pes-caprae*, *I. triloba* and *I. batatas*, as well as *I. biflora* and *I. aquatica*, were initially clustered into distinct clades. The phylogenetic relationships between *I. quamoclit* and *I. pes-caprae*, and between *I. triloba* and *I. batatas*, were shown to be closer than that between *I. biflora* and *I. aquatica*. Subsequently, these six *Ipomoea* species formed a larger clade, which then clustered with *I. trifida*, and finally grouped with *I. nil* (Figure 5).

## 4. Discussion

The plant mitochondrial genome has been established as a significant subject for research in plant phylogenetics and genetic diversity, due to its complex structural characteristics, dynamic evolutionary processes, and central role in energy metabolism and cellular regulation [7,24]. When compared to animal mitochondrial genomes, those of plants were demonstrated to exhibit greater diversity and complexity in terms of size, structure, repetitive sequence composition, and gene arrangement order [9,25]. In the present study, a systematic comparative analysis of the mitochondrial genomes was conducted for the first time across eight species within the genus *Ipomoea* (including *I. batatas*). Characteristics of the genus regarding mitochondrial genome structure, codon usage patterns, repetitive sequence distribution, selective pressures, and phylogenetic relationships were revealed, thereby providing a crucial foundation for understanding the evolutionary history and functional genomics of *Ipomoea* species.

The mitochondrial genome sizes of the eight *Ipomoea* species were found to range from 106.281 kb (*I. biflora*) to 296.652 kb (*I. aquatica*). The mitochondrial genome size of *I. batatas* was determined to be 257.880 kb, which was considered intermediate within this range. This size variation was largely consistent with the previously reported range for higher plant mitochondrial genomes (200 kb–2.34 Mb) [8], reflecting the high plasticity observed in plant mitochondrial genome size. The GC content of the *I. batatas* mitochondrial genome was calculated as 44.25%, which was similar to that of other *Ipomoea* species (43.89–44.82%), indicating a relative conservation in the base composition of mitochondrial genomes within this genus. However, significant interspecific variations were observed in the number of ORFs (298–326) and RNA editing sites (29–223). *I. trifida* was identified with the highest number of RNA editing sites (223), whereas only 29 sites were detected in *I. pes-caprae*, suggesting potential significant divergence in mitochondrial gene expression regulatory mechanisms among species [26]. Notably, an identical number of ORFs was not possessed by all *Ipomoea* mitochondrial genomes, and the functions of the majority of ORFs remain uncharacterized, a phenomenon similar to the uneven ORF distribution reported in Poaceae mitochondrial genomes [24]. Furthermore, a considerable number of RNA editing sites (215) were identified in the *I. batatas* mitochondrial genome, implying a potentially important role in post-transcriptional regulation, particularly in adaptation to various environmental stresses [15,27,28].

In higher plants, the types of protein-coding genes encoded by the mitochondrial genome are relatively conserved, but their number, location, and arrangement order often vary among different species, and even among different varieties of the same species [29]. The mitochondrial genomes of the *Ipomoea* species were found to encode 17 to 40 types of proteins. The highest number of protein types (40) was encoded by *I. trifida*, while the lowest (17) was encoded by *I. pes-caprae*. *I. batatas* was found to share most core protein-coding genes with the other species, including genes associated with complexes I–V, cytochrome c synthase genes (the *ccm* family), and ribosomal protein genes (*rpl*, *rps*). However, the absence of certain genes, such as *rps14*, was noted in *I. pes-caprae* and *I. quamoclit*, whereas this gene was present in the other six species, indicating a potential specific loss during the evolution of the genus. Similar phenomena have been reported in Poaceae, where *rps14* also exhibits high variability in some species [24]. Regarding gene copy number, single copies of *atp9* and *nad3* were identified in *I. batatas* and *I. trifida*, but these genes were completely absent in other species, suggesting independent gene duplication or loss events in certain lineages of *Ipomoea*. Such variations in gene copy number might be attributed to mitochondrial genome recombination or horizontal gene transfer [13,30,31], and could also be associated with species’ adaptive evolution. Analysis of codon usage bias revealed a general preference for A/U-ending codons, such as GUU (Val) and GCU (Ala), in the protein-coding genes of *I. batatas* and other *Ipomoea* species. GUU was the most preferred codon in *I. nil*, whereas GCU was the most preferred in the other species. Codons with low usage frequency included UAG (stop codon), UAC (Tyr), and CAC (His), with RSCU values ranging from 0.36 to 0.63. This codon usage bias was consistent with the A/T preference observed in other plants [24,32,33], possibly related to the high AT content of mitochondrial genomes, and reflecting optimization for translational efficiency and accuracy during evolution [23].

A high degree of diversity was observed in tRNA genes among the *Ipomoea* species. The greatest variety of tRNAs (27 types) was found in *I. quamoclit*, while the least (11 types) was found in *I. pes-caprae*. The *I. batatas* mitochondrial genome contained 20 types of tRNA, identical to *I. nil* and *I. trifida*. Ten tRNA types, including *trnC*, *trnF*, and *trnG*, were identified as the most conserved, being present in all species except *I. pes-caprae*, indicating their core functional role in the mitochondrial translation system of *Ipomoea*. In contrast, the distribution of *trnI* and *trnP* was highly variable; *trnP*, in particular, was present only in a few species, suggesting potential functional loss or replacement in specific lineages. Concerning copy number variation, *trnS* was present in three copies in *I. aquatica* and *I. biflora*, two copies in *I. nil* and *I. trifida*, and only one copy in *I. pes-caprae*. The highest copy number of *trnM* (4 copies) was observed in *I. quamoclit*, with fewer copies or absence in other species. These differences in tRNA copy number might influence mitochondrial translation efficiency, thereby affecting metabolic adaptation [34,35]. Repetitive sequences are considered major drivers of recombination and structural variation in plant mitochondrial genomes [8,36]. Three types of simple sequence repeats (SSRs) were identified in the *Ipomoea* species. P1 (mononucleotide repeats) were present in all species, while P3 (trinucleotide repeats) were detected only in *I. nil*. The total number of dispersed repeats was lowest in *I. triloba* (41) and highest in *I. trifida* (353). Certain types, such as DNA/MIRs and L3/CR1, were found only in specific species, indicating their potential involvement in lineage-specific genome reorganization events. Analysis of tandem repeats revealed that repeats in the 0–50 bp range were present in all species and were the most numerous. Repeats in the 51–100 bp and 101–200 bp ranges were most abundant in *I. quamoclit*. These long-fragment repeats might promote genome structure diversification through homologous recombination, consequently affecting gene arrangement and function [37]. Compared to Poaceae, the types of repetitive sequences in *Ipomoea* were relatively simplified, yet their role in genome structure evolution should not be overlooked. The evolutionary rates of mitochondrial protein-coding genes in *Ipomoea* species were assessed using the Ka/Ks ratio. The results indicated that Ka/Ks values were greater than 1 for 17 genes (*atp4*, *atp8*, *atp9*, *ccmFc*, *mttB*, *nad1*, *nad2*, *nad6*, *rpl5*, *rpl10*, *rps4*, *rps14*, *sdh4*), suggesting that these genes were under positive selection and may have played important roles in the adaptation of *Ipomoea* species to different ecological environments. Notably, the Ka/Ks values for *atp9* and *nad6* were 1.3230 and 1.3329, respectively, indicating they may have undergone adaptive evolution related to energy metabolism. The Ka/Ks values for the remaining 23 genes were all less than 1, with the lowest value (0.7135) observed for *nad3*, indicating this gene was under strong purifying selection, likely reflecting high functional conservation within the *NADH* dehydrogenase complex. This finding is consistent with the conclusion that most protein-coding genes in Poaceae are under purifying selection [24], underscoring the stability of core mitochondrial functional genes during evolution.

A maximum likelihood phylogenetic tree was constructed based on conserved mitochondrial protein-coding genes from the eight *Ipomoea* species. The tree revealed three distinct subclades: *I. quamoclit* and *I. pes-caprae* formed one subclade; *I. triloba* and *I. batatas* formed a second; and *I. biflora* and *I. aquatica* formed a third. The first two subclades were more closely related to each other. *I. trifida* and *I. nil* were positioned at the basal nodes of the tree, suggesting they may represent earlier diverging lineages within the genus. The phylogenetic relationships established in this study provide evidence at the mitochondrial genome level for the origin and dispersal of *Ipomoea* species and lay a foundation for future species identification and phylogenetic analysis using mitochondrial markers. Integrating analyses with chloroplast and nuclear genomic data in the future will help to further elucidate the evolutionary history of *Ipomoea*. In summary, this study represents the first systematic comparative analysis of the mitochondrial genomes of eight *Ipomoea* species, including the important crop *I. batatas*. Characteristics related to genome structure, gene content, repetitive sequences, codon usage, and selective pressure were revealed. The findings not only enrich the mitochondrial genome database for Convolvulaceae plants but also provide a theoretical basis for the evaluation of *I. batatas* germplasm resources, cultivar breeding, and evolutionary biology research.

## 5. Conclusions

Based on a comprehensive comparative analysis of the mitochondrial genomes of eight *Ipomoea* species, this study reveals significant interspecific variation in genome size, structure, gene content, and repetitive sequences. Key findings include a wide range of mitochondrial genome sizes (106–297 kb), divergent numbers of protein-coding genes (17–40) and tRNA types (11–27), and distinct patterns of codon usage bias and RNA editing. Evolutionary rate analysis (Ka/Ks) identified 17 protein-coding genes under positive selection, suggesting their role in adaptive evolution. Phylogenetic reconstruction provided robust insights into the evolutionary relationships within the genus. These results substantially enhance our understanding of mitochondrial genome evolution in *Ipomoea* and offer a valuable genomic foundation for future phylogenetic, functional, and breeding studies in this economically important genus.

## Figures and Tables

**Table 1:** Basic characteristics of the eight *Ipomoea* mitochondrial genomes.

Species	Mitogenome Size/kb	G + C Content/%	Number of ORF	Number of RNA Editing Site
*Ipomoea aquatica*	296.652	44.13	315	162
*Ipomoea quamoclit*	293.939	44.25	326	206
*Ipomoea biflora*	278.121	44.16	324	207
*Ipomoea batatas*	257.880	44.25	306	215
*Ipomoea nil*	265.768	44.45	324	182
*Ipomoea trifida*	264.698	44.15	310	223
*Ipomoea triloba*	160.302	43.89	298	165
*Ipomoea pes-caprae*	106.281	44.82	305	29

**Table 2:** Evolutionary rate analysis of PCGs in the eight *Ipomoea* mitochondrion.

Genes	Ka	Ks	Ka/Ks
*atp1*	0.9753	1.0788	0.9041
*atp4*	1.0303	0.9026	1.1414
*atp6*	0.9664	1.1245	0.8594
*atp8*	1.0080	0.9694	1.0398
*atp9*	1.0737	0.8116	1.3230
*ccmB*	0.9737	1.0837	0.8985
*ccmC*	0.9755	1.0680	0.9134
*ccmFc*	1.0048	0.9828	1.0223
*ccmFc1*	1.0273	0.9330	1.1011
*ccmFc2*	1.0055	0.9794	1.0266
*ccmFn*	1.0168	0.9448	1.0762
*Cob*	0.9903	1.0335	0.9582
*cox1*	0.9616	1.1156	0.8619
*cox2*	0.9602	1.1375	0.8441
*cox3*	0.9554	1.1314	0.8444
*matR*	0.9843	1.0488	0.9385
*mttB*	1.0448	0.8694	1.2018
*nad1*	1.0068	0.9804	1.0268
*nad2*	1.0092	0.9721	1.0381
*nad3*	0.9160	1.2834	0.7135
*nad4*	0.9887	1.0401	0.9506
*nad4l*	0.9580	1.1435	0.8378
*nad5*	0.9880	1.0427	0.9475
*nad6*	1.0672	0.8007	1.3329
*nad7*	0.9340	1.2797	0.7299
*nad9*	0.9757	1.0966	0.8897
*rpl5*	1.0187	0.9421	1.0813
*rpl10*	1.0335	0.8875	1.1644
*rpl16*	0.9217	1.2830	0.7184
*rps1*	0.9813	1.0552	0.9301
*rps3*	0.9973	1.0096	0.9878
*rps4*	1.00136	0.9948	1.0066
*rps7*	0.9592	1.1453	0.8374
*rps10*	0.9677	1.1224	0.8622
*rps12*	0.9916	1.0328	0.9601
*rps13*	0.9969	1.0147	0.9825
*rps14*	1.0288	0.8943	1.1504
*rps19*	0.9449	1.2618	0.7489
*sdh4*	1.0179	0.9451	1.0770

## Data Availability

The raw data supporting the conclusions of this article will be made available by the authors on request.
